# Critical analysis of psychosocial factors at work within the Risk Management Program of Regulatory Standard-1

**DOI:** 10.47626/1679-4435-2025-1425

**Published:** 2025-08-25

**Authors:** Sergio Roberto de Lucca, João Silvestre Silva Junior, Márcia Bandini

**Affiliations:** 1 Department of Public Health, Universidade Estadual de Campinas, Campinas, SP, Brazil; 2 Department of Legal Medicine, Bioethics, Occupational Medicine and Physical Medicine and Rehabilitation, School of Medicine, Universidade de São Paulo, São Paulo, SP, Brazil; 3 Centro Universitário São Camilo, São Paulo, SP, Brazil

**Keywords:** mental health, occupational risks, legislation, labor, saúde mental, riscos ocupacionais, legislação trabalhista

## Abstract

In the contemporary labor landscape, globalization, technological advancements, and the destabilization of working relationships have led to significant transformations, characterized by contract flexibilization, increased work intensity, and heightened psychological demands on workers. These changes have contributed to greater job insecurity and an increased prevalence of work-related illnesses, particularly mental health disorders and musculoskeletal conditions, which are among the leading causes of occupational absenteeism. The World Health Organization recognizes psychosocial factors at work as key determinants of occupational stress and occupational health outcomes. Inadequate management of these factors is associated with presenteeism, absenteeism, and adverse mental and physical health effects. In Brazil, although progress has been made, such as the incorporation of psychosocial factors into Labor Regulatory Standard-1, the issue remains insufficiently addressed. Assessments that rely exclusively on quantitative methodologies often fail to capture the subjective dimensions of workers’ experiences. This opinion article proposes a mixed-methods approach, integrating surveys and qualitative interviews, to comprehensively assess psychosocial factors in the workplace. A holistic evaluation, incorporating both objective and subjective aspects, is essential for an accurate understanding of these factors. Furthermore, the findings should inform the development of management strategies aimed at mitigating or eliminating workplace stressors that contribute to work-related illnesses. The adoption of inclusive policies, epidemiological monitoring, and participatory management frameworks can promote mental health improvements and the creation of safer and healthier work environments.

## Abstract (pt)

No mundo contemporâneo, o trabalho tem sido impactado por transformações decorrentes da globalização, de novas tecnologias e da precarização das relações de trabalho, incluindo a flexibilização dos contratos, a intensificação das atividades e a pressão psicológica sobre os trabalhadores. Esse cenário aumentou a insegurança e o adoecimento da força de trabalho, levando a uma maior incidência de transtornos mentais e distúrbios osteomusculares, que figuram entre as principais causas de afastamento laboral. A Organização Mundial da Saúde reconhece a influência dos fatores psicossociais no trabalho na gênese do estresse e no adoecimento dos trabalhadores. A gestão inadequada desses fatores resulta em desfechos negativos para a saúde dos trabalhadores. No Brasil, apesar dos avanços, como a inclusão dos fatores psicossociais na Norma Regulamentadora-1, ainda há uma abordagem limitada sobre o tema. Avaliações restritas à lógica quantitativa desconsideram a subjetividade inerente à experiência dos trabalhadores. Este artigo de opinião propõe a adoção de métodos mistos, combinando questionários e entrevistas, para capturar a complexidade dos fatores psicossociais no trabalho. As organizações devem considerar tanto os aspectos objetivos quanto os subjetivos na sua avaliação. Além disso, os resultados obtidos devem fundamentar um programa de gestão que contemple ações para mitigar ou eliminar os fatores psicossociais percebidos como desencadeantes de estresse e adoecimento. A adoção de práticas inclusivas, aliadas à vigilância epidemiológica e à gestão participativa, pode contribuir para a promoção da saúde mental e para ambientes de trabalho mais seguros e saudáveis.

## INTRODUCTION

Since the 1980s, transformations in capitalism have profoundly impacted the world of work. Globalization has introduced new production technologies and intensified the flow of financial capital across countries, fostering a new international division of labor and diverse forms of work organization. Increased flexibility in work activities and contracts, the intensification of work pace, and management strategies based on psychological pressure to enhance productivity have led to increase job insecurity and vulnerability among workers.^1^ In addition to traditional occupational accidents and diseases, new work-related illness have emerged, such as the global rise in musculoskeletal disorders^2^ and mental conditions, which are now among the leading causes of work absences and disability.^2,3^ In 1984, the International Labour Organization (ILO) and the World Health Organization (WHO)^4^ defined psychosocial factors at work (PFW) as resulting from the interactions between collective aspects, such as the work environment and organizational conditions, and the individual characteristics of workers, including skills, needs, culture, and personal circumstances. These factors can influence health, job performance, and overall job satisfaction.

The WHO considers the following elements as PFW: human resource management, alignment with organizational goals, communication and feedback mechanisms, autonomy, social support, workload and work pace, work environment and equipment, organizational culture, interpersonal relationships at work, role within the organization, career development, and work-home interface.^5^

Presenteeism (working while ill), absenteeism, and increased employee turnover can have socioeconomic consequences by reducing organizational productivity and imposing costs on society.^6^ In Europe, workplace PFW management programs have been proposed to mitigate work-related stress and illnesses.^5^ In Latin America, countries such as Chile, Colombia, Peru, Argentina, and Mexico have implemented regulations in this regard. In 2022, the ILO/WHO^7^ published the “Guidelines on mental health at work”, which include measures such as collective assessments of PFW and training for both managers and employees.

In Brazil, the recognition of PFW and work-related mental health issues has been largely neglected by the employment sector and representatives of neoliberal governments. The prevailing perspective still assumes that individual interventions aimed at strengthening workers’ resilience and coping skills are sufficient when provided by companies. Litle effort has been made to directly address PFW with the goal of fostering healthy and psychologically safe work environments and relationships.

From a regulatory perspective, PFWs only began to be included in Regulatory Standards (*Normas Regulamentadoras*, NR) issued by the Ministry of Labor and Employment (MTE) about a decade ago. Among them are NR-33 (Confined Spaces) and NR-35 (Work at Heights), which require that physical and mental fitness assessments of workers take psychosocial risk factors into account.^8,9^ In 2022, NR-5 expanded the responsibilities of the Internal Commitee for Prevention of Accidents and Harassment (*Comissão Interna de Prevenção de Acidentes e Assédio*, CIPA), establishing the obligation to adopt “measures aimed at preventing and combating sexual harassment and other forms of violence in the workplace.”^10^ In 2024, NR-1 (Occupational Risk Management, ORM) proposed the inclusion of “work-related psychosocial risk factors” in the occupational risk inventory of the Risk Management Program (PGR), alongside physical, chemical, biological, accident-related, and ergonomic hazards.^11^

This opinion article critically analyzes the relevance of the hazard and risk analysis model based on the probability and severity matrix, as proposed in NR-1, when applied to PFW.

## DISCUSSION

MTE Ordinance no. 1,419, of August 27, 2024,^11^ amends NR-1 as of May 26, 2025, establishing that ORM “must include risks arising from physical, chemical, and biological agents, accident risks, and risks related to ergonomic factors, including work-related psychosocial risk factors.” Additionally, it states that when assessing “the probability of occurrence of injuries or health impairments resulting from ergonomic factors, including work-related psychosocial risk factors,” organizations must also consider “the demands of the work activity and the effectiveness of the implemented prevention measures.”

The organization must determine the level of each occupational risk, which, in turn, must be established by combining the severity of potential injuries or health impairments with the probability of their occurrence. To this end, companies must select appropriate tools and techniques for the specific risk or circumstance being assessed and document the criteria used for grading severity and probability, risk levels, and the classification and decision-making criteria applied in the ORM.^11^

However, the proposed risk matrix model based on probability and severity originates from and is theoretically grounded in the field of occupational hygiene, where it has traditionally been applied to identify hazards and assess risks related to workers’ exposure to physical and chemical agents, measurable through occupational hygiene techniques.^12^ For a significant number of these agents, occupational exposure limits exist, incorporating quantitative assessment of concentration and/or dose exposure. These limits serve as reference values for evaluating workplace conditions and estimating occupational risk.^12^ Monitoring exposure over time alows the assessment of control measures’ effectiveness, particularly collective, process-based, or layout interventions.

Nevertheless, this objective and quantitative approach to risk and cause-effect evaluation does not directly apply to PFW, as these factors are intrinsically linked to the subjectivity and individuality of people, such as their relationships, expectations, and work-family experiences. Their nature is essentially qualitative, and their assessment requires methods, strategies, and tools that can capture workers’ perceptions of the factors that trigger stress, suffering, and illness. Therefore, PFW do not necessarily align within the traditional occupational health model.

Work is a central element in society. Beyond providing income for subsistence, it allows individuals to identify with their roles and feel socially valued, with the potential for recognition. For work to be emancipatory, promotive, and protective of mental health, it must allow individuals to exercise their potential and guarantee their subjectivity and autonomy through the freedom to perform their work well.^13^

In contrast, work that is dominated and lacks freedom leads to both physical and psychological strain. Domination over the worker is a determining factor in exhaustion, suffering, and illness, as it prevents the realization of subjectivity and the expression of freedom. The lack of autonomy and participation in the work process causes individuals to simply perform their tasks in a monotonous and repetitive manner, and this alienation can result in psychological suffering and illness.^14^ PFW are directly linked to both macroeconomic and microsocial contexts, as well as to the asymmetrical relations between capital and labor, which are influenced by the balance of power in each context.^15^ In this sense, atacks on workers can reach greater magnitude. An example of this is the deregulation of labor rights proposed by the labor reform and the flexibilization of certain NR between 2017 and 2022 to meet the demands of the employment sector, widely supported by ultraneoliberal governments.^16^

This anti-labor agenda has negatively impacted workers’ mental health, generating job insecurity – a situation that worsened during the pandemic, when unemployment soared, and social protection systems were weakened. Workplace management models based on psychological violence have become increasingly common, with psychosocial impacts and consequences for the workers’ well-being and work organization. In this context, the Brazilian Ministry of Health formally recognized the importance of PWF, by including them in the List of Work-Related Diseases (*Lista de Doenças Relacionadas ao Trabalho*, LDRT), which was expanded and updated in 2023.^17^ PFW were identified as aggravating and/or triggering factors for mental disorders and work-related suicides. Considering that the nature of PFW intertwines individual and collective dimensions, with an inseparable connection between living and work conditions, assessing PFW is more complex than what occupational health models can address. It is necessary to seek strategies that are appropriate to the significance of psychosocial factors and the risks of illness among workers.^18,19^ The scientific literature has well established the relationship between occupational stress and illness, primarily due to excessive psychological demands and low control at work,^20,21^ imbalance between effort and reward,^22^ and the concealment of emotions in the workplace.^23,24^

The practical application of these theoretical foundations has been studied since the 1980s, with the development of tools such as self-report questionnaires that include questions or items about psychosocial work dimensions. In this case, listening occurs indirectly, through a set of closed responses regarding the domains assessed in each theoretical model. The goal is to evaluate aspects such as the level of occupational stress and perceived illness among respondents, using a Likert-type scale within the spectrum of frequency or agreement for each question in the questionnaire. In this way, the response obtained for a qualitative atribute becomes objective and quantifiable. However, it is known that these tools do not capture the full subjective perception of the workers. Therefore, complementary qualitative assessments – such as focus groups, discussion rounds, and interviews – can help explore gaps not covered by the questionnaires regarding PFW. It is important to highlight that some psychosocial dimensions have a hierarchy, with emphasis on dimensions considered protective factors. Among them are social support, recognition, fair treatment, adequate remuneration, the quality of interpersonal relationships, cooperative work environments, and the level of trust in leadership – particularly in truthful and undistorted communication.^23^ These variables can also be identified in collectively administered questionnaires.

Self-report questionnaires should be administered in an environment that ensures confidentiality and anonymity for participants, as qualitative assessments may reveal psychological and subjective aspects that, although individual and unique, complement the initial findings of the quantitative diagnosis.^15,25^ The main limitation of the risk concept when applied to PFW is that harmful agents are traditionally analyzed in isolation.^15^ Thus, using the term “psychosocial risk factor” as employed in NR mistakenly equates the evaluation process of PFW to that of measurable risk factors, such as chemical or physical agents. The analysis of PFW requires a broader and longitudinal approach to the interactions between quantitative and qualitative aspects.

Despite any criticism, it is a fact that NR-1 will require an evaluation based on the probability and severity of health effects. To contribute, we offer some suggestions that can guide this process:

- Since the nature of PFW is intrinsic to the organization of work, all workers should be considered “exposed,” regardless of their position or the activities they perform.
- The collective evaluation of PFW^25,26,27,28,29^ should be conducted using validated instruments and must be preceded by the explicit consent of workers and their representatives, with voluntary participation and a guarantee of anonymity for participants. The results should be analyzed according to the specificities of the economic activity and its epidemiological profile.
- The organization’s active epidemiological surveillance should be conducted longitudinally, based on the LDRT, considering diseases and health issues within the collective of workers, regardless of their employment relationship.
- Data on long-term absenteeism should include information on the granting of temporary social security disability benefits (work-related and non-work-related), permanent disability pension, and accident compensation provided by the Brazilian Social Security National Institute. Short-term absences should also be analyzed.
- Information on presenteeism can contribute to assessing the impact of PFW on workers’ ability to perform their jobs.
- In addition to mental disorders, data on work-related accidents and other health conditions, such as repetitive strain injuries and work-related musculoskeletal disorders, can be used to assess the impacts of PWF.
- The number of cases of suicidal ideation, atempts, and completed suicides – whether occurring inside or outside the workplace – must be considered.
- The number of reported cases of moral and/or sexual harassment, registered in the organization’s internal grievance system or external entities, including worker representation bodies, must be analyzed.

However, none of these measures will be effective if organizations do not genuinely commit to revising their management practices and models, promoting active and constructive participation from those who work within them. The goal must be to create work environments and processes that are psychologically safe, capable not only of protecting mental health but, perhaps more importantly, of promoting it. Ultimately, work should be emancipatory, allowing individuals to realize their full potential and contribute to generating wealth for the country, rather than merely fueling an endless pursuit of profit at any cost.

## References

[1] Antunes R, Praun L (2015). A sociedade dos adoecimentos no trabalho. Serv Soc Soc.

[2] Fernandes RCP, Carvalho FM, Assunção AA, Silvany Neto AM (2009). Interactions between physical and psychosocial demands of work associated
to low back pain. Rev Saude Publica.

[3] Seligmann-Silva E, Bernardo MH, Maeno M, Kato M (2010). O mundo contemporâneo do trabalho e a saúde mental do
trabalhador. Rev Bras Saude Ocup.

[4] International Labour Organisation (ILO) (1986). Psychosocial factors at work: recognition and control.

[5] World Health Organization (2008). PRIMA-EF: guidance on the European framework for psychosocial risk management: a
resource for employers and worker representatives [Internet].

[6] Silva Jr JS, Fischer FM (2014). Long-term sickness absence due to mental disorders is associated with
individual features and psychosocial work conditions. PLoS One.

[7] World Health Organization (WHO), International Labour Organization (ILO) (2022). Mental health at work.

[8] Brasil, Ministério do Trabalho e Emprego (2020). NR-33 segurança e saúde nos trabalhos em espaços confinados
[Internet].

[9] Brasil, Ministério do Trabalho e Emprego (2023). NR 35: trabalho em altura [Internet].

[10] Brasil, Ministério do Trabalho e Previdência, Gabinete do Ministro (2022). Portaria nº 4.219, de 20 de dezembro de 2022.

[11] Brasil, Ministério do Trabalho e Emprego, Gabinete do Ministro (2024). Portaria nº 1.419, de 27 de agosto de 2024.

[12] Serviço Social da Indústria (SESI) (2022). Manual técnico da metodologia SESI de avaliação de riscos
ocupacionais [Internet].

[13] Dejours C (2004). Subjetividade, trabalho e ação. Rev Prod.

[14] Seligmann-Silva E (2011). Trabalho e desgaste mental: o direito de ser dono de si mesmo.

[15] Pereira ACL, Souza HA, Lucca SR, Iguti AM (2020). Fatores de riscos psicossociais no trabalho: limitações para
uma abordagem integral da saúde mental relacionada ao trabalho. Rev Bras Saude Ocup.

[16] Krein D, de Oliveira RV, Filgueiras A (2019). As reformas trabalhistas: promessas e impactos na vida de quem
trabalha. Cad CRH.

[17] Brasil, Ministério da Saúde, Gabinete da Ministra (2023). Portaria GM/MS Nº 1.999, de 27 de novembro de 2023.

[18] Lucca SR (2017). Fatores psicossociais e risco de adoecimento no trabalho. Rev Laborativa.

[19] Fischer FM (2012). Relevância dos fatores psicossociais do trabalho na saúde do
trabalhador. Rev Saude Publica.

[20] Karasek Jr RA (1979). Job demands, job decision latitude and mental strain: implications for job
redesign. Am Sci Q.

[21] Karasek R (2008). Low social control and physiological deregulation— the
stress-disequilibrium theory, towards a new demand-control model. Scand J Work Environ Health.

[22] Siegrist J (1996). Adverse health effects of high-effort/low-reward conditions. J Occup Health Psychol.

[23] Pejtersen J, Kristensen T, Borg V, Bjorner J (2010). The second version of the Copenhagen Psychosocial Questionnaire (COPSOQ
II). Scand J Prim Health Care.

[24] Fernandes C, Pereira (2016). A exposição a fatores de risco psicossocial em contexto de
trabalho: revisão sistemática. Rev Saude Publica.

[25] Lucca SR, Sobral R (2017). Aplicação de instrumento para o diagnóstico dos
fatores de risco psicossociais nas organizações. Rev Bras Med Trab.

[26] Alves MGM, Chor D, Faerstein E, Werneck GL, Lopes CS (2009). Job strain and hypertension in women: Estudo Pró-Saúde
(Pro-Health Study). Rev Saude Publica.

[27] Araujo TM, Graça CC, Araujo E (2003). Estresse ocupacional e saúde: contribuições do Modelo
Demanda-Controle. Cienc Saude Coletiva.

[28] Gonçalves JS, Moriguchi CS, Chaves TC, Sato TO (2021). Cross-cultural adaptation and psychometric properties of the short version
of COPSOQ II-Brazil. Rev Saude Publica.

[29] Chor D, Werneck GL, Faerstein E, Alves MGM, Rotenberg L (2008). The Brazilian version of the effort-reward imbalance questionnaire to
assess job stress. Cad Saude Publica.

